# Microneedles and Their Application in Transdermal Delivery of Antihypertensive Drugs—A Review

**DOI:** 10.3390/pharmaceutics15082029

**Published:** 2023-07-27

**Authors:** Ramsha Khalid, Syed Mahmood, Zarif Mohamed Sofian, Ayah R. Hilles, Najihah Mohd Hashim, Yi Ge

**Affiliations:** 1 Department of Pharmaceutical Technology, Faculty of Pharmacy, Universiti Malaya, Kuala Lumpur 50603, Malaysia; ramshaamaheen@gmail.com (R.K.); ms_zarif@um.edu.my (Z.M.S.); 2 INHART, International Islamic University Malaysia, Jalan Gombak, Kuala Lumpur 53100, Malaysia; ayah.hilles90@gmail.com; 3 Department of Pharmaceutical Chemistry, Faculty of Pharmacy, Universiti Malaya, Kuala Lumpur 50603, Malaysia; najihahmh@um.edu.my; 4 Center for Natural Products Research and Drug Discovery (CENAR), Universiti Malaya, Kuala Lumpur 50603, Malaysia; 5 School of Pharmacy, Queen’s University Belfast, Belfast BT9 7BL, UK

**Keywords:** microneedle, transdermal, hypertension, polymeric nanoparticles, lipid nanoparticles

## Abstract

One of the most cutting-edge, effective, and least invasive pharmaceutical innovations is the utilization of microneedles (MNs) for drug delivery, patient monitoring, diagnostics, medicine or vaccine delivery, and other medical procedures (e.g., intradermal vaccination, allergy testing, dermatology, and blood sampling). The MN-based system offers many advantages, such as minimal cost, high medical effectiveness, comparatively good safety, and painless drug application. Drug delivery through MNs can possibly be viewed as a viable instrument for various macromolecules (e.g., proteins, peptides, and nucleic acids) that are not efficiently administered through traditional approaches. This review article provides an overview of MN-based research in the transdermal delivery of hypertensive drugs. The critical attributes of microneedles are discussed, including the mechanism of drug release, pharmacokinetics, fabrication techniques, therapeutic applications, and upcoming challenges. Furthermore, the therapeutic perspective and improved bioavailability of hypertensive drugs that are poorly aqueous-soluble are also discussed. This focused review provides an overview of reported studies and the recent progress of MN-based delivery of hypertensive drugs, paving the way for future pharmaceutical uses. As MN-based drug administration bypasses first-pass metabolism and the high variability in drug plasma levels, it has grown significantly more important for systemic therapy. In conclusion, MN-based drug delivery of hypertensive drugs for increasing bioavailability and patient compliance could support a new trend of hypertensive drug delivery and provide an alternative option, overcoming the restrictions of the current dosage forms.

## 1. Introduction

**Figure 1:**
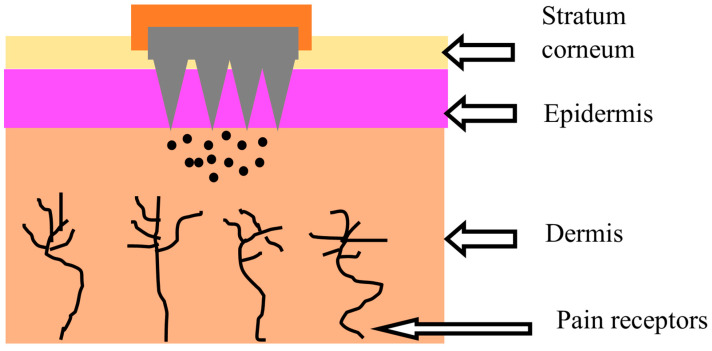
Microneedle-based drug delivery through the skin.

Hypertension (HTN) is defined as a pathological disorder that is characterized by elevated blood pressure (BP), i.e., the systolic around 140–150 or above [1]. The World Health Organization (WHO) has identified HTN as one of the leading causes of mortality and morbidity globally, accounting for nearly 9 million deaths yearly [2]. Substantial scientific studies have linked high blood pressure (BP) to various disorders, such as cardiovascular disorders and heart failure [3]. Hypertension is also considered a significant risk factor for the development of angina pectoris, chronic kidney disease (CKD), diabetes miletus (DM), and atrial fibrillation [4,5]. Hypertension and diabetes are considered to often coexist together. The risk of developing diabetes, mostly type 2, is higher in patients with hypertension than in healthy people. Following that, several clinical trials and epidemiological studies revealed a link between antihypertensive drugs (e.g., thiazide diuretics or beta-blockers) and the development of diabetes (type 2). Some reported studies have found that beta-blockers, in particular, appear to enhance the incidence of diabetes in hypertensive patients [6]. Apart from this, the elevated risk linked to high BP can mostly be considerably reduced with antihypertensive therapy, which reduces both associated target organ damage and BP [7,8]. The first-line treatment of HTN could be chosen from the following classes: angiotensin receptor blockers (ARBs), angiotensin-converting enzyme inhibitors (ACE inhibitors), calcium channel blockers (CCBs), and thiazide-type diuretics. Each antihypertensive class lowers the risk of cardiovascular events [9]. It is challenging for hypertensive patients to follow their therapeutic regimens using traditional dosage forms, including tablets, capsules, and injections [10]. The pediatric population suffering from hypertension has trouble swallowing medications and is allergic to needles [5]. Most antihypertensive drugs are given in the form of tablets; nevertheless, tablets have several disadvantages, including gastrointestinal discomfort, drug degradation in the stomach, irregular absorption, and pre-systemic drug metabolism, eventually leading to lower bioavailability [10,11]. Such issues can be solved to some extent by administering antihypertensive drugs via transdermal microneedle (MN)-based drug delivery systems [12]. The restrictions of the oral and injectable routes are bypassed with the MN-based transdermal drug delivery system, as the needles are non-invasive, low-cost, and easy to use [13,14,15,16]. They are micron-sized needles that are less than 1000 µm in size and can penetrate the principal barriers of the skin that inhibit drug molecules’ transport through the stratum corneum without causing pain (Figure 1) [15,17]. MN systems can provide precise drug localization with reduced dosing frequency and improve patient compliance through the convenience of administration and better biodistribution with efficacy [18]. A significant problem for the therapeutic application of antihypertensive medications is their limited aqueous solubility.

Likewise, few hypertensive drugs (e.g., valsartan, benidpine, and felodipine) belong to the Biopharmaceutical Classification System (BCS) class II; they possess poor aqueous solubility and high drug permeability. The goal is to improve the solubility of poorly aqueous-soluble drugs to increase their bioavailability [19]. Drugs with low water solubility are formulated in non-aqueous carriers such as lipid-based systems and delivered through MNs directly into the skin. This approach encapsulates and solubilizes poorly soluble drugs. MNs allow drugs to bypass the stratum corneum to enable direct access to the underlying tissue and bypass the need for drug solubility in water [20]. High drug permeability refers to the ability of the drug to pass through biological barriers, like tissues or cell membranes, in order to reach its targeted site of action [21]. For antihypertensive drugs to exhibit higher drug absorption, both their solubility and permeability must be higher. In the case of antihypertensive drugs, higher permeability is desirable as it allows for efficient absorption and distribution throughout the body to exert their therapeutic effect [22]. Recently, numerous cutting-edge research studies on microneedles reported that they could significantly increase transdermal drug delivery across the human skin safely [23,24,25]. This review exclusively focuses on the emerging field of MN-mediated delivery of antihypertensive drugs. The aim of this paper is to provide an overview of recent advancements in utilizing MN technology for the efficient administration of antihypertensive drugs.

In addition, we also discuss other factors, such as the potential benefits, limitations, and prospects of MN-based delivery of antihypertensive drugs compared to the traditional route. We emphasize the existing literature on MNs targeting hypertension and their types involving manufacturing. The present manuscript is the first to cover hypertension and MNs. It will benefit the researchers and clinicians working on MNs and hypertension.

## 2. Overview of Hypertension

### 2.1. Hypertension and Different Treatment Strategies for Hypertension

**Figure 2:**
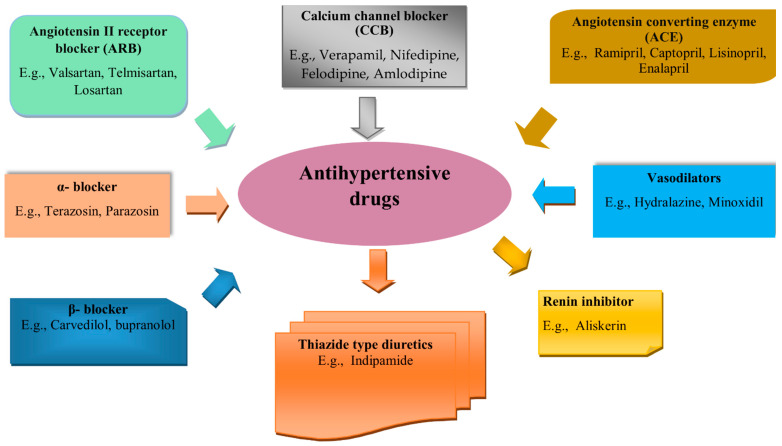
Classification of drugs used in hypertension.

Hypertension, often known as arterial hypertension, is a chronic medical disorder characterized by abnormally elevated pressure in the arteries. The systolic and diastolic measurements of blood pressure refer to either the contraction of the heart muscle, called systole, or the relaxation of the heart muscle, named diastole [26,27]. According to the American College of Cardiology (ACA) and the American Heart Association (AHA), a normal blood pressure range is 100–139 mmHg systolic and 60–89 mmHg diastolic [28]. When the blood pressure is regularly at or higher than 140/90 mmHg, hypertension is presumably present [28,29]. Hypertension is a significant risk factor for disorders like renal disease and myocardial infarction (MI) [30]. Blood pressure (BP) levels directly impact the likelihood of developing disorders like coronary artery disease and stroke [31]. Hypertension treatment has been designated a global health priority [32,33]. Primary (essential) hypertension and secondary hypertension are two types of hypertension. Primary (essential) or idiopathic hypertension refers to hypertension with no secondary causes, such as renal failure, monogenic forms, and renovascular disease, which accounts for nearly 95% of cases globally [27]. Essential hypertension is a heterogeneous condition, with various people experiencing different causes of high blood pressure, whether environmental or genetic. Secondary hypertension is caused by multiple factors, including renal, vascular, and endocrine [26]. Although lifestyle interventions can help manage blood pressure and reduce health issues, therapy is typically required for individuals with insufficient dietary and lifestyle interventions [26,34]. The most important and extensively used antihypertensives are thiazide diuretics, angiotensin-converting enzyme (ACE) inhibitors, calcium channel blockers (CCBs), β-blockers, and angiotensin II receptor antagonists (ARBs) (Figure 2). Individual patient characteristics influence antihypertensive drug selection. Risk factors like advanced age and arrhythmia linked to elevated BP must all be taken into account, while patient tolerance, comorbidity, and drug interactions all play a role in the selection of antihypertensive drugs [31].

### 2.2. Etiology and Risk Factors of Hypertension

Blood pressure elevations have been associated with various etiological factors as potential causes that can lead to the onset of hypertension [35,36,37]. Etiological factors, e.g., low intake of sodium and potassium, high sodium intake, low intake of fruits and vegetables, being overweight, lack of physical activity, and tobacco and alcohol use, can lead to hypertension. Metabolic disorders such as hyperinsulinemia and insulin resistance are also etiological factors that lead to hypertension [38]. 

### 2.3. Problems Concerning Oral Drug Administration of Antihypertensive Drugs

The most common way to achieve a systemic effect is through the oral administration of drugs. Compared to other administration methods, drug delivery through the oral route is the most desired and extensively used method, with painless administration, patient adherence, and so on [39]. Nonetheless, problems related to the physical properties of active substances, such as limited water solubility, instability, and low permeability, all lead to irregular and low oral bioavailability, making oral administration difficult [40]. Extreme pH, delayed attainment of pharmacodynamic effect, poor intestinal permeability, unpredictable bioavailability, sustained toxicity, dosing inflexibility, dose dumping, and CYP 450-mediated enzymatic metabolism are all factors that prevent the drug from being administered orally [41]. The aqueous solubility of an orally taken drug determines how well it dissolves in the gastrointestinal tract. Hydrophobic oral medications have poor absorption, dosage proportionality, and unwanted side effects [42]. 

Oral formulations (e.g., tablets) are usually the primary choice for drug administration because of their comparative ease of preparation and use [43]. There are numerous drugs available that can treat hypertension in traditional dosage forms. Several studies have been published on the oral administration of antihypertensive drugs [44,45,46]. The traditional oral dosage of antihypertensive drugs typically involves immediate-release formulations, which often require multiple doses per day to maintain therapeutic drug levels, leading to fluctuations in blood pressure and decreased patient compliance. Compared to traditional oral administration, the controlled release of oral antihypertensive drugs can be administered once or twice daily with comparable therapeutic efficacy and fewer adverse reactions than standard formulations. Several antihypertensive drugs have been developed as controlled-release formulations, including calcium channel blockers, β-blockers, and angiotensin-converting enzyme inhibitors. These controlled-release formulations are typically designed to be taken less frequently, making it easier for patients to comply with their therapeutic regimen. Controlled-release formulations of calcium channel blockers, such as diltiazem, nifedipine, and verapamil, provide a sustained drug release over 24 h, reducing the dosing frequency and minimizing side effects [47]. A controlled-release tablet of β-blockers, e.g., propranolol hydrochloride, was prepared to reduce dosing frequency and improve patient compliance [48,49]. Controlled-release formulations of ACE inhibitors, such as perindopril, were also formulated to provide a sustained release of the drug over 24 h [50].

**Figure 3:**
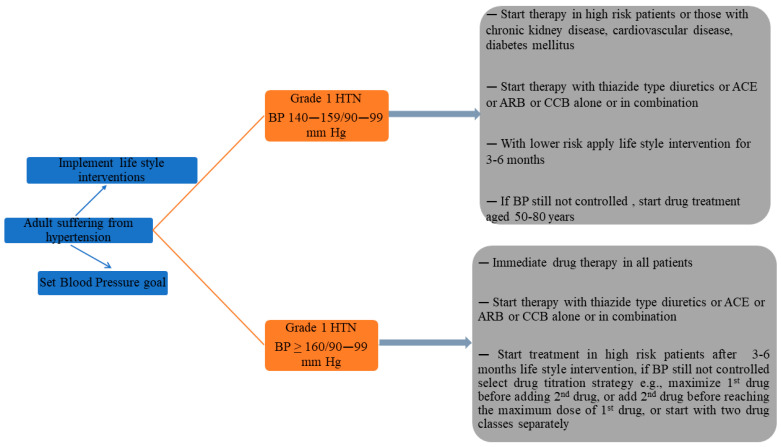
Algorithm for hypertension management (JNC-8 guidelines).

A summarized list of antihypertensive drugs showing physicochemical and metabolic profiles is shown in Table 1. The treatment algorithm for hypertensive patients is shown in Figure 3.

## 3. Overview and Classification of Microneedles

**Figure 4:**
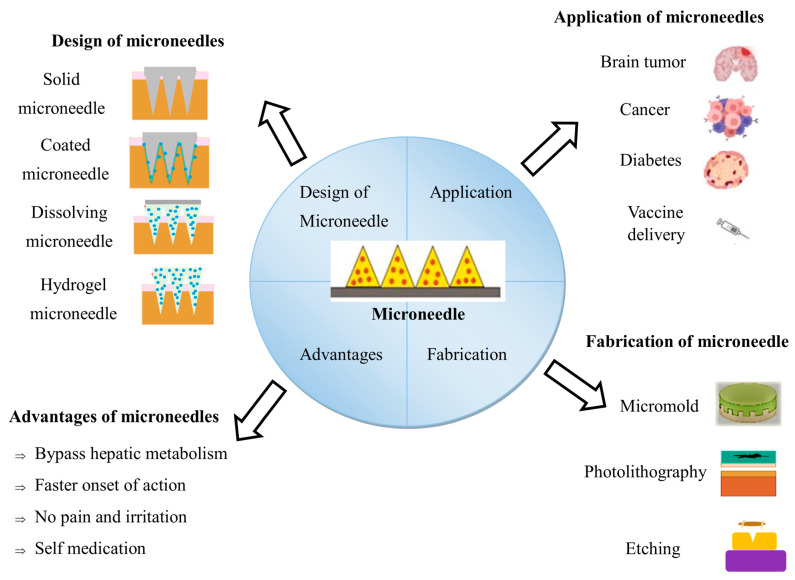
Summary of microneedles and their applications.

**Figure 5:**
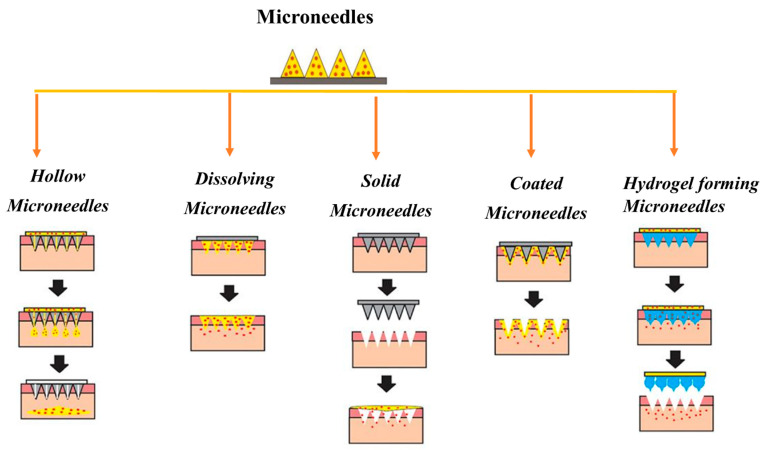
The drug delivery through different types of microneedles, i.e., hollow microneedles (poke and flow method), dissolving microneedles (poke and release method), solid microneedles (poke and patch method), coated microneedles (coat and poke method), and hydrogel-forming microneedles (poke and release method) as shown by Al-Japairai et al. 2022) [99].

MNs are medical devices with a single or a group of needles with a diameter of a few micrometers that transport drug components to the outermost layers of the skin [77,78]. For transdermal drug delivery, a needle with a length of up to 1000 µm is long enough to pass through the stratum corneum and release the drug into the dermis [79,80]. MNs benefit from reducing tension or anxiety caused by needle phobia, vasovagal reactions, and pain experienced when a traditional needle is used [81,82]. MNs are fabricated from various materials, e.g., silicon, metal, and glass. Depending on the manufacturing procedure, the needles’ shapes might range from cones to square pyramids [83]. MNs must be powerful enough to pierce the skin without producing blood [84]. To release the entrapped pharmaceutical cargo and prevent sharp waste, the MNs should preferably decompose in the human skin [85]. Due to their unique characteristics and avoidance of hazardous waste sharps, MNs have advantages over oral and other drug delivery methods [86,87]. Pediatrics and geriatrics who have trouble swallowing would benefit from this system [86]. Depending on the desired drug delivery mechanism, these can be categorized into different types: silicon MNs, dissolving MNs, solid MNs, polymer MNs, glass MNs, hydrogel-forming MNs, ceramic MNs, metal MNs, coated MNs, sugar MNs, and hollow MNs [88,89,90,91,92,93,94,95] (Table 2) (Figure 4 and Figure 5). During the early development stage, most of the MNs were made of silicon [96]. Some metals, including titanium, stainless steel, nickel, and others, have mechanical qualities that are well integrated, such as high strength and toughness, which can protect MNs against mechanical failure. Furthermore, metal MNs can be produced at a lower cost than silicon MNs. Metal MNs will also generate biohazardous tip waste [97]. The most promising materials for MN manufacturing are polymers. They can be adaptable, easily accessible, cost-effective, biocompatible, and have sophisticated features, such as built-in controlled release mechanisms [94]. Polymers have higher toughness than silicon-like brittle materials, allowing polymer MNs to prevent brittle breakage upon insertion into the skin. Polymer MNs rarely cause severe side effects because most polymers are biocompatible. It is worth noting that, because most polymers have a low melting temperature, several manufacturing techniques, like micromolding, are suitable for the low-cost mass production of MNs. As a result, polymers are gaining popularity and are seen as potential materials for MN manufacturing [97]. MN technology has steadily advanced for the past four decades. MNs can transfer vaccines, insulin, and other pharmaceutical dosage forms via the skin, according to numerous preclinical investigations and a small number of clinical trials [98].

## 4. Fabrication and Current Status of Microneedles

### 4.1. Microneedle Design and Fabrication

**Figure 6:**
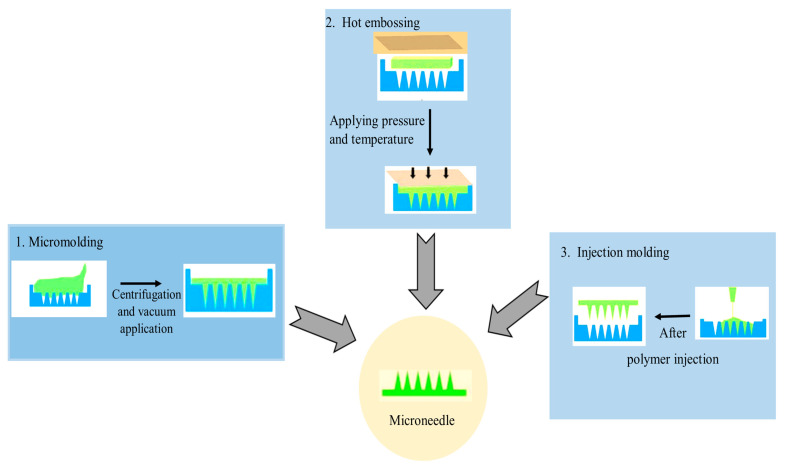
Various casting and molding techniques are used for the preparation of MNs ((Figure reproduced with modification (Modified form Bhatnagar and group, 2019 [119]).

When designing MNs for skin penetration, there are a few important factors to consider: (a) physical characteristics, such as hollow, solid, side-opened, beveled, and conical tipped; (b) material choice; (c) geometric characteristics, such as diameter, length, shape, and tip size; (d) array layout; (e) fabrication feasibility [113]. Some MN fabrication technologies include wet chemical etching, injection molding, laser drilling, reactive ion etching, hot embossing, drawing lithography, and lithography with electroforming (Table 3) (Figure 6). To date, silicon deep reactive ion etching (DRIE), micromolding, and photolithography are the most extensively used production techniques for the fabrication of microneedles [113,114]. Microelectromechanical systems are commonly used to fabricate microneedles. The basic procedure for making MNs can be broken down into three steps: (a) deposition, making a deposit, (b) patterning, creating patterns, and (c) etching or engraving [115]. There are various ways to manufacture microneedle devices, including surface/bulk micromachining, injection molding, reactive ion etching, isotropic chemical etching, and so on [115].

**Table 3:** Overview of key microneedle fabrication.

Material	Fabrication Method	Comments	Advantage	Disadvantage	References
Metal	Drawing lithograph; lithography, electroplating and molding (LIGA); laser drilling; electrodeposition; photochemical etching; electroplating	Porous structure long microneedles causing pain during administration	Good mechanical properties, high fracture resistance, robust and difficult to break, biocompatible	Cause an allergic reaction, costly startup	[100,113,116]
Silicon	Silicon deep reactive ion etching (DRIE); micromolding; Photolithography; LIGA (which uses deep X-ray lithography)	Better biocompatibility but brittle and prone to shatter in use	Desirable sizes that are sufficiently flexible to be manufactured	Fabrication takes a long time and is an expensive procedure and causes skin fractures	[100,113,117]
Polymer	Casting; photolithography; micromolding;	Biocompatible polymers, painless microneedles	Outstanding biocompatibility, limited toxicity, and affordable	Limited strength of microneedles	[100,113,118]
Ceramic	Micromolding; lithography	Transfers the geometric shape master pattern to the substrate’s surface	Exhibits resistance to chemicals and compression	Limited tension strength of microneedles	[100]

#### 4.1.1. Silicon MN Fabrication

**Figure 7:**
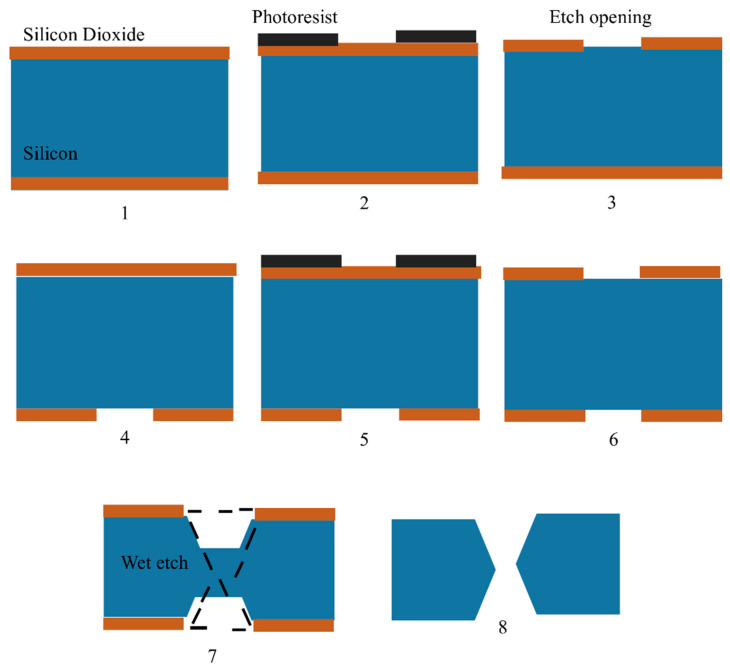
Figure showing steps for in-plane silicon microneedle fabrication as shown by Howells, et al., 2022 [121]. Silicon wafers are represented in blue, silicon dioxide is shown in brown and photoresist is shown in black.

Silicon MNs have been the most prevalent form of MNs, manufactured using microfabrication techniques that require complex multistep processes and expensive tools explicitly made for the microelectronics industry. Wet and dry etching, which are subtractive methods, are the most commonly used methods for fabrication [113]. Before creating the MNs from the front side of the silicon wafer, the microneedle channels were first etched from the backside of the wafer using a combination of isotropic and anisotropic etching [120]. The fabrication of silicon MNs is shown in Figure 7. Silicon MNs are fabricated using silicon wafers with double-sided polish and a thickness of 300 µm. Thermal silicon dioxide is generated on the wafers’ front and back. Fabrication of silicon MNs consists of seven steps, which are as follows: (1) A 300 µm wafer is coated with 2.5 µm of silicon dioxide via chemical vapor deposition, (2) the silicon-dioxide hard mask is then etched with the device pattern using the inductively linked plasma etching technique, (3) this is followed by the wafer flipping and patterned utilizing alignment marks on the backside by photolithography, (4) inductively linked plasma etching is used on the device pattern etched into the silicon dioxide hard mark, (5) the entire wafer is then immersed in a 44% potassium hydroxide solution to etch it, (6) the etching process takes five hours, (7) finally, the equipment is taken out of the potassium hydroxide solution [121]. (Figure 7).

#### 4.1.2. Polymer MN Fabrication

**Figure 8:**
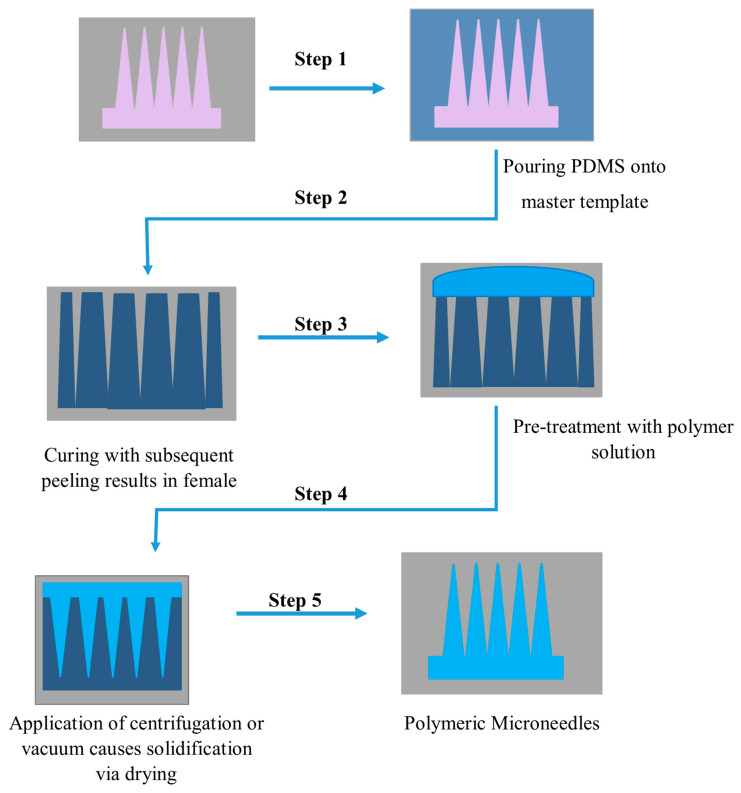
Schematic illustration of polymeric microneedle fabrication via polydimethylsiloxane PDMS micro molding (Figure modified from Wang M et al., 2017 [122]).

Polymer materials are gaining popularity because of their outstanding mechanical qualities, biocompatibility, and biodegradability. Polymer microneedles also have a lower fabrication cost than silicon microneedles. Photolithography, micromolding, and micromachining are increasingly becoming the preferred production processes for developing polymer microneedles [113,114]. Photolithography is the process of chemically modifying a liquid material and then exposing it to short-wavelength light to polymerize it. The resist is sprayed onto the substrate surface for patterning and later exposed to light (typically UV light) using a projection stepper, followed by wet development to form a resist pattern. This procedure requires a photosensitive material like polymethylmethacrylate for fabrication [113]. The fabrication of MNs by micromolding is shown below in Figure 8 [113]:

### 4.2. Diverse Application of MNs in Advanced Drug Delivery

When MNs are designed to fit the qualities of drugs, such as their polarity and pharmacological characteristics, they are regarded as an appropriate method for delivering various drug moieties via continuous transdermal delivery. Typically, drugs are inserted into the microneedle matrix or deposited onto the surface of the microneedle tips. The drugs can sometimes be preloaded into nanoparticles encased in the microneedle matrix to regulate the medication release profile [123]. The latest advancements in drug delivery via MNs include small molecule delivery, insulin delivery, cosmeceuticals, and cancer treatment [124]. Drugs possessing molecular weights below 500 Da can penetrate the skin passively, but the penetrated amounts are insufficient to produce therapeutically effective doses. As a result, polymeric MNs have been used to improve the transdermal delivery of drugs with small molecular sizes [122,125].

### 4.3. Mechanism, Pharmacokinetics, and Insertion Behavior of MNs

**Figure 9:**
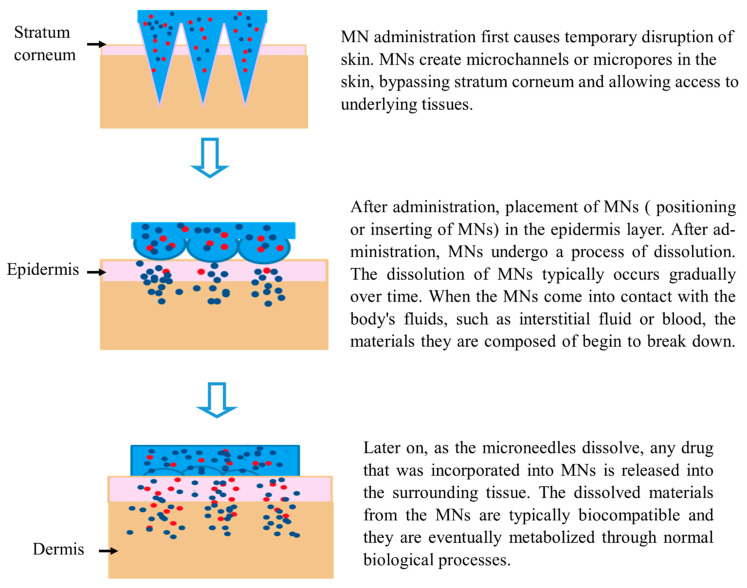
Mechanism of action of MNs. Blue color represents a fast drug releasing mechanism, while red color indicates a slow-release mechanism.

Hundreds of MNs, each less than 1 mm long, are arranged in an MN array to deliver medication to the skin. An MN patch is developed when an MN array is connected to an adhesive backing to help its adhesion to the skin [126]. The drug is deposited into the dermis layer of the skin, where it can easily reach its intended target site as part of the drug delivery process, resulting in temporary mechanical disruption of the skin (Figure 9). Additionally, MNs provide microscale drug delivery channels while bypassing still-functioning blood vessels and nerve terminals in the epidermis and dermis. As a result, drug delivery efficiency is improved, and greater doses and medicines with larger molecular sizes can be administered without difficulty [127]. For example, biodegradable polymers have been utilized to fabricate polymeric MN-containing drugs inside a polymer matrix. These MNs puncture the skin; the polymers disintegrate, releasing the medications into the bloodstream and causing a therapeutic reaction at the site of action [79].

Following MN application, pharmacokinetics explains how the body reacts to a therapeutic moiety and how it flows through, out of, and into the body (including metabolic changes, absorption, and distribution mechanisms) [124]. The pharmacokinetic profile of MNs can be influenced by several factors, such as the rate at which the polymer matrix dissolves, the total drug dose injected into the MN, and the enzymatic degradation of the drug into the skin [97]. Lee’s research team employed a dissolving polymeric MN composed of carboxymethylcellulose and gelatin to deliver insulin to diabetic rats. They found that the pharmacokinetic parameter area under the curve (AUC) value increased following the MN application. Polymeric MNs for insulin administration showed adequate pharmacokinetics compared to typical hypodermic injections [128]. These findings suggest that MNs could be extremely effective transdermal drug delivery systems. MNs offer much potential for the quick, painless, and convenient administration of medications by fulfilling pharmacokinetic requirements.

The insertion behavior of MNs into the skin can also be influenced by the structure and mechanical properties of the skin, as determined by Kong and Wu [129]. Factors, such as the effects of the MN length on effective drug delivery and associated pain, must be considered while designing MNs [130]. The lowest insertion force is always expected because it naturally signifies less pain and invasiveness [131].

### 4.4. Advantages of MNs for Drug Delivery, Patient Monitoring, Diagnostics, and Vaccine Delivery

MNs have potential advantages over conventional hypodermic needles for drug and vaccine delivery. MNs are designed with unique dimensions to avoid stimulating nerves and causing patient discomfort. MNs have the potential to be administered without clinical expertise, as they are in the form of affordable disposable patches to improve the pharmacokinetic profile of therapeutic component delivery. For example, disposable MN patches could reduce the transmission of HIV by encouraging the self-administration of tests and treatments [113]. Some other advantages of MN-based drug delivery are: faster healing at the injection site than a hypodermic needle, decreased microbial penetration, the MN punctures only the epidermis, specific skin areas can be targeted for desired drug delivery, dose reduction, and the drug delivery rate can be controlled more effectively by this drug delivery system [132].

Nowadays, MNs have been employed as a diagnostic aid in managing diseases. The traditional methods for blood withdrawal are characterized by discomfort and fear among patients; this can be avoided by employing MNs through their painless biofluid withdrawal method. With their ease of usage, MNs represent a unique tool for detecting a wide range of biomarkers from skin interstitial fluid, including small molecule metabolites, nucleic acids, proteins, and even cells. Recent studies revealed the status of diseases (cancer, diabetes, arthritis, etc.) by analyzing blood and tissue using hollow MNs or quantum dots [133].

MNs have been widely investigated in the past years to enhance transdermal drug delivery. Recently, researchers have realized the potential of MNs for enhancing patient monitoring. Several methods of patient monitoring with MNs have been proposed, including using solid arrays for pretreatment before fluid collection, hollow microneedle arrays for fluid collection and subsequent off-site analysis, and integrated options, eliminating the need for fluid removal. Regardless of the strategy, the MN device must successfully and repeatedly penetrate without fracture and provide precise measurements of the target analytes to be a useful alternative to existing practices. When looking into MN platforms for minimally invasive patient monitoring, there are many factors to consider, and many different strategies have been taken into account [134].

The use of MNs in vaccination is particularly appealing since it provides the anticipated benefits of simplifying vaccine administration, enhancing patient compliance, and permitting vaccine targeting to the skin. It is well recognized that administering vaccines through the skin has immunologic benefits over doing so by intramuscular injection. Still, there have not been any straightforward, dependable techniques for doing so. This constraint can be overcome by using MNs, including hollow MNs for intradermal injection and solid MN patches. The use of MNs for vaccine delivery has received the most research attention due to these opportunities [85].

There are different routes for drug delivery, which include oral, intravenous, transdermal patches, etc. The oral route is the most traditional and convenient for patients, with acceptable ease of administration but limited bioavailability. The oral route adversely affects long-term medications because it affects crucial organs, including the liver and kidneys [100]. However, a transdermal patch requires the drug to cross the stratum corneum barrier, thus showing less bioavailability. The transdermal patch can improve drug permeation by adding a permeation enhancer, but up to a minimal extent. The hypodermic needle goes deep into the dermis, where pain receptors are present. As a result, it can deliver 90–100% of the loaded drug, but because it is painful, it results in poor patient compliance. MNs bypass the stratum corneum barrier and deliver the drug directly into the epidermis and dermis layers, delivering 100% of the loaded drug without causing pain [79].

### 4.5. MN-Mediated Antihypertensive Agents and Some Reported Nanoparticle-Based Delivery Systems

The link between cardiovascular disease and high blood pressure is widely established in the scientific literature. Hypertension can lead to kidney failure and coronary artery disease if left untreated. Hypertension is still a life-threatening medical issue, despite recent breakthroughs in hypertension research and therapies [1]. As a result, it is imperative to develop and test innovative hypertensive medicines to enhance patients’ long-term clinical care and results. Several hypertension animal models have been developed recently to facilitate in vivo testing of both treatment methods and medication efficacy [135].

Recently, MNs have been used as an alternative drug delivery system to deliver antihypertensive drugs across the skin barrier painlessly. MN-based drug delivery reduces the risk of adverse effects associated with oral antihypertensive medications. Additionally, MNs can be used to deliver sustained-release formulations of antihypertensive drugs, which can help improve patient compliance and reduce dosing frequency. Overall, MN-based drug delivery of antihypertensive drugs has the potential to be a safe and effective treatment option for patients with hypertension. Recently, much research has been going on to develop and optimize the delivery system for different antihypertensive drugs [24,136]. Some research utilizing MN-based drug delivery of antihypertensive drugs is discussed here.

An antihypertensive dissolving MN was developed utilizing concurrent medicine, e.g., sodium nitroprusside (SNP) in combination with sodium thiosulfate (ST). Dissolvable MNs were fabricated by centrifugal casting using SNPs and ST. SNPs were stably packaged into microneedles and rapidly delivered into the systemic circulation using this approach. Antihypertensive microneedle treatment (aH-MN) reduced blood pressure quickly and significantly. It met the clinical standards for hypertensive emergency blood pressure treatment. Concurrent delivery of ST successfully decreased the negative effects (e.g., organ damage) caused by SNP ingestion. This research demonstrated an effective and user-friendly biodegradable patch for the controllable delivery of drugs in antihypertensive therapy [23,137]. Ahad et al. formulated eprosartan mesylate-loaded transferosome gel to study skin permeation. The pharmacodynamic study showed better management of hypertension after the application of transferosome gel as compared to an oral formulation. An MN roller, e.g., a dermaroller, was utilized on rats’ skin to increase the permeation enhancement of the drug. The transdermal flux of the eprosartan mesylate from transferosome gel across rat skin improved when pretreated with an MN [138]. Table 4 summarizes the published research data in which hypertensive drugs are formulated using an MN-based approach.

#### 4.5.1. Calcium Channel Blockers (CCB)

Calcium channel blockers are generally well absorbed following oral administration. However, all calcium channel blockers undergo significant first-pass metabolism, dramatically lowering oral bioavailability. Traditional dosage forms have significant drawbacks, including hepatic first-pass metabolism, a high incidence of side effects due to varied absorption profiles, a higher frequency of administration, and poor patient compliance. Attempts have been undertaken to develop novel drug-delivery systems for various calcium channel blockers, including microneedle-based delivery systems, to overcome conventional drug delivery’s disadvantages [31]. In 2014, Kaur et al. fabricated MN rollers and solid stainless steel MNs using amlodipine besylate and verapamil hydrochloride. Passive penetration of verapamil and amlodipine across the skin was observed to be very low. To improve the percutaneous penetration of these antihypertensive drugs, the effect of the fabricated MNs was observed. It was mentioned that the percutaneous flux of amlodipine besylate after using stainless steel MNs was significantly enhanced.

Moreover, the transcutaneous flux of verapamil across MN-roller-treated porcine skin was also increased. The author has claimed that stainless steel solid MNs and MN rollers increased percutaneous penetration of verapamil hydrochloride and amlodipine besylate significantly [139]. Another study was conducted to investigate the effect of MN parameters like shape, length, density, and type on the enhancement of transdermal permeation using an antihypertensive drug, e.g., amlodipine. The study showed that MN application enhanced amlodipine transdermal permeation, and MN geometrical parameters play a significant role in such permeation enhancement [140]. Sardesai et al. formulated a bio-responsive MN with an advanced drug delivery system by incorporating nifedipine, a cardiodepressant, and diltiazem, a vasodilator, for effective synergism to treat hypertension. The study revealed that formulated MN systems co-delivering antihypertensive drugs significantly and substantially lowered blood pressure compared to conventional drug delivery systems [141]. Another study was conducted in which MN rollers were developed for the transdermal permeation of two antihypertensive drugs, e.g., diltiazem hydrochloride and perindopril erbumine. This study showed increased transdermal permeation using an antihypertensive drug-loaded MN roller [1]. Alkilani et al. conducted a study to develop and evaluate a transdermal delivery system of amlodipine besylate loaded with biodegradable polymeric nanoparticles for sustained drug delivery. A study claimed transdermal delivery of amlodipine besylate in a controlled manner by incorporating biodegradable polymeric nanoparticles in hydrogel MN [142]. Kolli et al. used solid maltose MNs made by micromolding techniques to instantly deliver nicardipine hydrochloride across rat skin. MNs create microchannels in the skin, which have been shown to improve the transdermal delivery of nicardipine hydrochloride [108].

#### 4.5.2. Angiotensin-Converting Enzyme Inhibitors (ACE)

Angiotensin-converting enzyme inhibitors have been used for many years to manage heart failure and hypertension. ACE inhibitors prevent the conversion of angiotensin I to angiotensin II, lowering blood pressure by reducing blood vessel tension and blood volume. ACE inhibitors are currently administered either intravenously or orally. Many researchers have investigated MN-based drug delivery as an advanced alternative approach for ACE inhibitor delivery over the years [143]. Stainless steel MN arrays and rollers were developed to study the transcutaneous flux of captopril and metoprolol tartrate across the skin. There was a significant enhancement in the transdermal flux and permeation across the skin for captopril and metoprolol tartrate following MN arrays and rollers [144].

#### 4.5.3. Angiotensin II Receptor Antagonists (ARB)

Angiotensin II receptor antagonists are a group of pharmaceuticals that modulate the renin–angiotensin–aldosterone system. ARBs may be used alone or in combination with other medications, frequently diuretics. When patients cannot tolerate ACE medication for their hypertension, ARBs are usually preferred. ARBs show extensive first-pass metabolism and poor availability when given orally. MN-based drug delivery can avoid poor bioavailability and other limitations of ARB. Several studies have been investigated to develop and study the effect of MN-based delivery of ARBs [52]. Huang et al. fabricated an MN patch system composed of dissolving gelatin and starch and loaded with losartan. The dissolving MN patches effectively penetrated the stratum corneum and exhibited strong mechanical strength.

The study showed that the developed MN patch system increased the drug delivery efficiency of losartan upon dissolution of gelatin and starch [145]. Pineda-Álvarez et al. formulated biodegradable polymeric MN arrays loaded with losartan potassium nanoparticles as a novel pharmaceutical drug delivery intended to be used for blood pressure control. The study revealed that the optimal formulation of the MN array with nanoparticles constitutes the most appropriate option for the transdermal delivery of losartan [146]. Enggi et al. developed valsartan transdermal gel to overcome valsartan’s poor absorption and low bioavailability. Moreover, solid MNs were combined with transdermal gel to improve the percutaneous permeation of valsartan across the stratum corneum. The study revealed that using MNs significantly enhanced valsartan permeation across the skin and significantly impacted the treatment of hypertension [147]. Another research study was conducted to determine the release of valsartan from transdermal patches and the effect of solid MN on its permeation. The transdermal patch consists of polyethylene glycol 400 as a permeation enhancer. The study revealed that incorporating polyethene glycol enhanced valsartan’s permeation across the skin. Notably, a combination of transdermal patches and MNs significantly improved the permeation of valsartan [148]. Likewise, Nirmayanti et al. developed a thermosensitive hydrogel for sustained transdermal delivery of valsartan, further enhanced by solid MNs.

The formulation, containing hydrogel and solid MNs, increased the permeation of valsartan. The transdermal delivery of valsartan using this combinational approach improved the bioavailability of the drug as compared to oral delivery [149]. Moreover, another study described the simultaneous delivery of a combination of three drugs (aspirin, lisinopril dihydrate, and atorvastatin calcium trihydrate) using a dissolving polymeric MN system. Studies showed that it is possible to deliver a combination of drugs from a single-dissolving MN array [150]. Almazan et al. developed a losartan potassium patch using MN for the treatment of hypertension and its complications, where components of the matrix system permit a controlled drug release. This matrix system increases the bioavailability of losartan and avoids multiple doses. The authors claimed that multiple doses, dose-associated side effects, and gastric irritations could be avoided, making the matrix system more convenient for patients [151].

#### 4.5.4. β-Blockers

β-blockers are one of the widely prescribed cardiovascular drugs that are mainly used in conventional dosage forms. These dosage forms have a number of drawbacks, including the need for more frequent administration and low patient compliance. Essentially, efforts have been undertaken to develop novel delivery systems for β-blockers, including transdermal drug delivery, to circumvent the drawbacks of traditional drug delivery. However, notable laboratory research projects have been carried out globally to examine skin penetration and create transdermal formulations of beta-blockers [59,152]. He et al. fabricated dissolving MNs of propranolol hydrochloride (HCL) by using matrix materials such as hyaluronic acid and polyvinyl pyrrolidone. The study revealed that employing dissolving MNs significantly enhanced the permeability and skin retention of propranolol [153]. Pawar et al. studied the effect of drug lipophilicity on MN-mediated iontophoretic delivery across the skin. The study claimed that the lipophilic properties of drugs play an essential role in the electrically assisted transdermal delivery of drugs across the skin. The study showed that the delivery rate of hydrophilic drugs, atenolol and sotalol, using a combination of iontophoresis and MN was much higher than that of lipophilic drugs (acebutolol and propranolol) [154]. Ita et al. used stainless steel MNs and gold–titanium MN rollers to transdermally deliver β-blockers, e.g., atenolol and bisoprolol, across porcine ear skin. The study concluded that MNs significantly enhanced the percutaneous penetration of atenolol and bisoprolol across the skin [155]. Jiang et al. developed a transdermal patch combining binary ethsomes and MNs to improve the poor bioavailability of carvedilol. The reported study indicates that MN-based transdermal drug delivery patches significantly reduced the fluctuation of carvedilol concentration in plasma, thus reducing blood pressure for longer. The MN transdermal drug delivery patch showed a promising prospect for improving the bioavailability of carvedilol as compared to the oral route for treating chronic diseases such as hypertension [156].

#### 4.5.5. Diuretics

Diuretics are the second most commonly prescribed class of antihypertensive drugs, and thiazide diuretics have been prescribed more than all other antihypertensive drugs combined. The necessity of diuretics for all patients, but especially for those with salt-sensitive and resistant hypertension, has been emphasized in the hypertension guidelines. Diuretics work by increasing the excretion of sodium and water from the body, thereby reducing fluid volume and blood pressure [157]. While the concept of an MN-based delivery of diuretics for hypertension is promising, it is important to note that further research is needed to explore its feasibility, safety, and effectiveness. There is an extensive amount of research ongoing; however, there are currently very few studies available.

Abu-Much et al. fabricated biodegradable polymeric MNs of furosemide for controlled drug delivery. The study revealed that MNs exhibited an initial burst release followed by a sustained release of furosemide. Self-administered furosemide MNs can open new avenues to overcome the limitations associated with them and increase patient compliance [158].

**Table 4:** A summary list of published microneedle studies categorized according to their therapeutic application and BCS drug classification.

Drugs	Class	MN Type	BCS Drug Classification	Therapeutic Area	Comments	References
Captopril, metoprolol tartrate	ACE inhibitor and β-blocker	Stainless steel microneedle rollers and arrays	BCS class III, BCS class I	Hypertension	Significant enhancement in transdermal flux after the application of microneedle	[144]
Sodium nitroprusside	Vasodilator	Dissolvable microneedle patch	-	Hypertension	Rapid and potent BP reduction was observed	[23]
Propranolol hydrochloride	β-blocker	Dissolving microneedles	BCS class I	Infantile hemangioma	Significantly increased the permeability and skin retention, enhancing dermal delivery	[153]
Diltiazem hydrochloride, perindopril erbumine	Calcium channel blocker and ACE inhibitor	Microneedle rollers	BCS class I, BCS class III	Hypertension	Increase in transdermal penetration by 113.59-fold	[1]
Nifedipine, diltiazem	Calcium channel blocker	Bio-responsive microneedles	BCS class II and BCS class I	Hypertension	Significantly reduced the mean blood pressure, system co-delivers the drug	[141]
Verapamil, amlodipine	Calcium channel blocker	Stainless steel solid microneedles and microneedle rollers	BCS class I for both	Hypertension	Increased percutaneous penetration	[139]
Hydrophilic drugs (atenolol, sotalol) and lipophilic molecules (propranolol, acebutolol)	β-blockers	Microneedle-mediated iontophoretic delivery	BCS class III BCS class I BCS class I BCS class III		Increase permeation rate	[154]
Lisinopril dihydrate, atorvastatin, aspirin	ACE inhibitors, statins, salicylates	Microneedle arrays	BCS class I BCS class III BCS class II	Cardiovascular disease	Combined drug delivery from a single-dissolving MN array	[150]
Amlodipine	Calcium channel blocker	Polymeric microneedle	BCS class I	Hypertension	Enhanced transdermal permeation	[140]
Nicardipine hydrochloride	Calcium channel blocker	Solid microneedle	BCS class II	Hypertension	Improved transdermal delivery of nicardipine	[108]
Amlodipine besylate	Calcium channel blocker	Hydrogel microneedle	BCS class I	Hypertension	Transdermal delivery of amlodipine in controlled manner	[142]
Losartan potassium	Angiotensin receptor blocker	Polymeric microneedle	BCS class III	Hypertension	Improved transdermal delivery	[146]
Losartan	Angiotensin receptor blocker	Dissolving microneedle patch	BCS class III	Skin disease	Enhanced drug delivery efficiency	[145]
Valsartan	Angiotensin receptor blocker	Solid microneedle	BCS class II or III	Hypertension	Improved drug permeation and significant impact on hypertension	[147]
Valsartan	Angiotensin receptor blocker	Solid microneedle	BCS class II or III	Hypertension	Enhanced drug permeation using combination of transdermal patch and microneedle	[148]
Valsartan	Angiotensin receptor blocker	Solid microneedle	BCS class II or III	Hypertension	Improved bioavailability of valsartan	[149]
Atenolol, bisoprolol	β-blockers	Stainless steel microneedles and gold titanium microneedle rollers	BCS class III BCS class I		Enhanced percutaneous penetration of drugs	[155]
Carvedilol	β-blockers	Microneedle	BCS class II	Hypertension	Improved bioavailability of carvedilol	[156]
Furosemide	Loop diuretics	Polymeric microneedle	BCS class IV		Sustained release of furosemide	[158]

### 4.6. MNs for the Transdermal Delivery of Hypertensive Drugs: Critical Attributes and Upcoming Challenges

Several drugs are available in conventional dosage forms to treat hypertension, but most antihypertensive drugs are poorly water soluble and therefore have low bioavailability. These drugs are Pgp (P-glycoprotein) substrates and have problematic characteristics, such as a short half-life, poor intestinal permeability, and high dose frequency [159]. Antihypertensive drugs with poor absorption rates are ideal for MN-based drug delivery. MNs overcome the issues related to the oral delivery of antihypertensive drugs by increasing percutaneous absorption as the drug passes directly into the systemic circulation. MNs create micron-sized pores in the skin and disrupt only the stratum corneum and epidermis, but do not reach nerve fibers or blood vessels in the dermis. These MNs can deliver hypertensive drugs transdermally, potentially providing more effective treatment with fewer side effects than traditional oral medications [139].

Critical attributes of MNs for the transdermal delivery of hypertensive drugs include the material of the needles, the number of needles per patch, the length and diameter of the needles, and the method of drug loading onto the needles. The needles must be long enough to penetrate the skin’s stratum corneum but not so long that they reach the nerve fibers and cause pain. The diameter of the needles should be small enough to minimize discomfort but large enough to allow for efficient drug delivery. The number of needles per patch should be optimized for the desired drug dose and delivery rate. The material of the needles should be biocompatible and durable enough to withstand insertion into the skin [15].

One of the upcoming challenges associated with this approach is ensuring consistent drug delivery across various skin types and conditions. Skin thickness, hydration level, and other factors can affect the penetration of the needles and the delivery of the drug. Another challenge is optimizing the drug formulation and loading method for each specific drug to ensure optimal delivery efficiency and stability. Likewise, challenges with respect to dose delivery are also apparent, as relatively low doses can be used with the MNs. Furthermore, the repeated use of MNs at the exact location may cause swelling and skin irritation [103].

### 4.7. MNs Bypass First-Pass Metabolism and High Variability in Drug Plasma Levels of Antihypertensive Drugs

MN-based drug administration bypasses first-pass metabolism and reduces the variability in drug plasma levels by delivering the drug directly into the systemic circulation through the skin [132]. When taken orally, drugs are absorbed into the bloodstream through the digestive system and then metabolized by the liver before reaching the rest of the body. This process, known as first-pass metabolism, can reduce the bioavailability of drugs and result in variable plasma levels. First-pass metabolism limits oral absorption [160]. In contrast, MNs deliver drugs directly through the skin, bypassing the liver and the gastrointestinal tract, increasing the bioavailability of drugs, and resulting in more consistent plasma levels, leading to more effective treatment with fewer side effects. Furthermore, MN-based delivery offers a convenient, painless, and discreet alternative to traditional oral medications, increasing patient compliance and improving treatment outcomes [79]. Many patients with hypertension struggle to take their medications regularly due to side effects, difficulty swallowing, or forgetfulness. MNs can overcome these problems. Overall, MN-based drug administration has the potential to revolutionize the delivery of hypertensive drugs by increasing bioavailability, reducing side effects, and improving patient compliance [136].

### 4.8. Patents on MNs

As MN applications are a novel method for the transdermal delivery of therapeutic agents, many patent applications have been filed. Since hollow MNs may deliver more medicine than other microneedles, their design and delivery are the main topics of most of these patent applications. Hollow MN technology has received many patents due to its advantage of being able to administer a higher drug volume than other MNs [161,162]. However, there is limited data available on antihypertensive drug-loaded MN patents. Yet, a patent application, US7627938B2, titled “Tapered hollow metallic microneedle array assembly and method of making and using the same”, was published using the antihypertensive drug clonidine [163]. Another patent application, WO 2020/250210 A1, titled “Microneedles, and methods for the manufacture thereof”, was filed using antihypertensive drugs carvedilol and nifedipine as the pharmaceutical ingredients. The patent stated that MNs of the invention may incorporate up to 99% of the drug or more. The legal status of this patent is still pending [164].

Similarly, another patent, CN 110584633, titled “Real-time hypertension diagnosis and treatment integrated system capable of realising controllable medicine release”, was filed but is still pending. Moreover, patent EP 2005990 A2, titled “Microneedle Device And Transdermal Administration Device Provided With Microneedles”, stated that low molecular weight drugs, such as bisoprolol, can sustain the effect of the drug for a long period of time. Its legal status is still active [165]. It is worth noting that research and development in the field of MN-based drug delivery systems is constantly evolving, and there may have been advancements since then. There is still not much research on MN-based delivery of antihypertensive drugs. This area needs to be addressed, and further research work is required.

## 5. Conclusions

MNs are considered innovative drug delivery systems with unique benefits. They are the perfect platform for pharmaceutical and biological applications since they have improved pharmacokinetics, safety, and efficacy when delivering active substances to the targeted spot. They have offered groundbreaking solutions for the delivery of active therapeutic ingredients employing MNs in life-threatening conditions. All application areas, such as illness detection, disease therapy, immune-biological, dermatological, and aesthetic applications, have seen significant advancements. MNs play a crucial role in achieving a drug release profile; selecting the appropriate material, manufacturing procedure, needle geometry, and design is vital. Clinical trials on MNs have been conducted, demonstrating the scientific community’s significant interest in using devices for various therapeutic purposes. As a result, specific MN devices have made it to the commercial market. The development of these minimally invasive devices would provide a variety of therapeutic opportunities for drug delivery via buccal, oral, and ocular routes. Some studies have reported that MN-based delivery of antihypertensive drugs improves the transdermal delivery of these drugs. However, further research is needed to evaluate the effectiveness of MN in treating hypertension. They can be successfully adapted for clinical application after a greater grasp of the challenges and constraints of the MN-based delivery of antihypertensive drugs is developed. Ultimately, MN-based drug delivery holds the promise of revolutionizing the treatment of hypertension and improving patient outcomes. The current review paper sheds light on difficulties associated with oral drug delivery of antihypertensive medications, MN-based delivery of antihypertensive drugs, and their effect on hypertension.

## Figures and Tables

**Table 1:** Physicochemical and metabolic profile of oral antihypertensive drugs.

Class	Drug	BCS Class	Metabolism	Solubility	t_1/2_	Log P	Oral Bioavailability	References
Calcium channel blocker	Verapamil	BCS class I	Extensively metabolized by CYP2C8, CYP2C18, and CYP2C9 hepatic first-pass metabolism,	7 mg/mL	2–7 h	3.8	10–20%	[31,51]
Calcium channel blocker	Nifedipine	BCS class II drug	Hepatic metabolism by CYP3A4 high first pass metabolism	20 µg/mL	2 h	2.20	45–56%	[31,51]
Calcium channel blocker	Amlodipine	BCS class I	Extensive hepatic Metabolized by CYP3A4	75.3 µg/mL	30–50 h	2.22	64%	[31,51]
Calcium channel blocker	Felodipine	BCS class II	Inclusively metabolized by CYP3A4	7.15 µg/mL	7–21 h	4.36	15%	[31,51]
Angiotensin II receptor blocker	Valsartan	BCS class II	CYP2C9	<0.1 mg/mL	7.5 h	5.8	<25%	[51,52]
Angiotensin II receptor blocker	Losartan	BCS class III	CYP2C9, CYP3A4	3.3 mg/mL	1.5–2 h	4.5	25–35%	[52,53,54]
Angiotensin II receptor blocker	Telmisartan	BCS class II	CYP2C8, CYP2C9, and CYP2J2	9.9 μg/mL	24 h	7.7	42–58%	[55,56,57]
Beta-blocker	Carvedilol	BCS class II	CYP1A2, CYP3A4, CYP1A1, CYP2D6, CYP2E1, CYP2C9	0.583 µg/mL	7–10 h	4.1	25–35%	[51,58,59]
Beta-blocker	bupranolol	BCS class II	CYP2D6 first-pass metabolism (>90%)	-	2–4 h	2.9	10%	[59,60]
Renin inhibitor	Aliskiren	BCS class III	CYP3A4- mediated hepatic metabolism	122 mg/mL as salt	24 h	2.45	2.5%	[51,61]
Angiotensin-converting enzyme inhibitor	Ramipril	BCS class I	Carboxylesterase	3.5 mg/mL	13–17 h	0.92	55–65%	[52,62,63]
Angiotensin-converting enzyme inhibitor	Captopril	BCS class III	-	125–160 mg/mL	1–3 h	1.02	65%	[52,64]
Angiotensin-converting enzyme inhibitor	Lisinopril	BCS class III	-	0.45 mg/mL	12 h	1.2	25%	[52,65]
Angiotensin-converting enzyme inhibitor	Enalapril	BCS class III	Carboxylesterase	-	11–14 h	0.19	40–60%	[52,62]
Thiazide-like diuretics	Indapamide	BCS class II	CYP3A4	-	14 h	2.52	100%	[52,62]
Alpha-blocker	Terazosin	BCS class III	-	-	12 h	2–3	Completely absorbed	[66,67,68]
Alpha-blocker	Prazosin	BCS class II	-	0.2–1.6 mg/mL	2.9 h	0.173	56.9%	[69,70,71,72]
Vasodilators	Hydralazine	BCS class III	CYP1A2		2–7 h	0.83	30–50%	[73,74,75]
Vasodilators	Minoxidil	BCS class II	-	2.2 mg/mL	4.2 h	0.6	90%	[74,76]

**Table 2:** A detailed list of advantages, disadvantages, and applications of various MNs.

MN Type	Characteristics/Method of Delivery	Advantages	Disadvantages	Application	Material	References
Hollow microneedle	Control release of the drug throughout the time pressure drives the flow through needle	High dose of drug solution, proper materials used to attain hydrophilic behavior, constant flow rate, dose accuracy, easy to formulate	Needle design requires great care, to withstand flow pressure, require durable material for preparation, require prefilled syringes, blocking the narrow channels	Disease diagnosis	Silicon	[100,101,102,103]
Coated microneedle	Low dose of the encapsulated drug, coating drug release	Rapid drug delivery through the skin, potent drugs requiring low doses increase the permeability of drugs, stability of drug, mechanical strength	Prone to infection and drug loss during fabrication, damaged needles irritate, expensive manufacturing method	Drug and vaccine delivery	Silicon	[100,103,104]
Solid microneedle	Channels created in the skin cause drugs to penetrate the lowest layers of the skin, sharper tip, adequate mechanical strength	Allows more drugs to pass into the skin, easy to formulate, can be formulated from a range of materials, increase the permeability of drugs	To prevent the possibility of infections, microincisions required to be stitched up, fracture of micron-sized needles under the skin, restricted availability of drug surface area available	Drug delivery	Silicon Metal Polymer	[100,103,105,106]
Dissolving microneedle	Macromolecules release rapidly, releasing the drug by dissolving under the skin	Ease of administration for patients, easy manufacturing	Requires technical expertise to manufacture, utilization of biodegradable materials, dissolving takes time	Drug and vaccine delivery	Polymer	[100,103,107,108]
Porous microneedle	Loading of drug achieved by different pore sizes; during manufacturing, porosity can be achieved	Higher capability of drug loading, the simplest method employed for fabrication	Lower ability to penetrate the skin	Disease diagnosis	Stainless steel, Titanium	[109,110,111]
Hydrogel	Sustained drug release achieved by a minimally invasive device	Significantly improved biocompatibility, biodegradability, tolerability, affordability, controlled release of drug	Lower mechanical strength	Drug delivery	Polymer	[110,111,112]

## Data Availability

Not applicable.
